# Hybrid CNN-LSTM-GNN Neural Network for A-Share Stock Prediction

**DOI:** 10.3390/e27080881

**Published:** 2025-08-20

**Authors:** Junhao Dong, Shi Liang

**Affiliations:** Department of Mechanical Engineering, University of Hong Kong, Hong Kong; junhaodong@connect.hku.hk

**Keywords:** stock prediction, Pearson and IG weighted selection, GNN, CNN, LSTM, China’s A-shares mark

## Abstract

Optimization of stock selection strategies has been a topic of interest in finance. Although deep learning models have demonstrated superior performance over traditional methods, there are still shortcomings. For example, previous studies do not provide enough explanation for feature selection and usually use features such as closing price directly to make predictions; for example, most studies predict the trend of multiple stock indices or only individual stocks, which is difficult to be directly applied to actual stock selection. In this paper, a multivariate hybrid neural network model CNN-LSTM-GNN (CLGNN) for stock prediction is proposed, in which the CNN and the LSTM modules analyze the local and the whole, respectively, while the multivariate time series GNN module is added to explore the potential relationships between the data through the graph learning, graph convolutional, and temporal convolutional layers. CLGNN analyzes the potential relationships between the data based on the returns to classify stocks, and then develops a stock selection strategy, and directly outputs the returns and stock codes. In this paper, a hybrid filter approach based on entropy and Pearson correlation is proposed for feature selection, and experiments are conducted on all stocks in the CSI All Share Index (CSI); the results show that among multiple models, the returns obtained when the features of daily return, turnover rate, relative strength index, volume, and forward adjusted closing price are used as inputs are all the highest, and the return obtained by CLGNN is even higher than that of the other models (e.g., TCN, Transformer, etc.).

## 1. Introduction

In the domain of financial investment, investors have a range of options and channels at their disposal to generate income. The stock market, a major financial market, has been a subject of considerable interest from investors and scholars. Scholars have been trying to figure out how to predict the returns of different stocks in order to make the best investment portfolio and obtain the highest return. However, due to the dynamic, nonlinear, unstable, and complex nature of financial markets, there has been a long debate in the past about whether stocks are predictable or not. The Efficient Market Hypothesis (EMH), proposed by Fama [1] on the basis of theories such as the stochastic wander theory, posits that in the special case where the stock price reflects all the available information, and only in the very short term of a weakly efficient market, the historical price information is of some significance. Since then, this theory has gradually gained much support (e.g., In *A Random Walk Down Wall Street*, Burton Malkiel posits that stock prices exhibit a “random walk” tendency in the short term, thereby precluding the possibility of predicting returns that exceed the market mean [2]). However, this perspective is not fully accepted among scholars in the field.

Narasimhan Jegadeesh and Sheridan Titman argued that the stock market has some degree of predictability in the short run, and that buying stocks that have trended up in the past and selling those that have trended down in the past can result in significant short-term gains within a year [3]. Donald B. Keim and Robert F. Stambaugh’s study demonstrates the efficacy of a range of ex ante variables in predicting changes in market risk premiums and providing insights into future asset returns [4]. Lakonishok, Shleifer, and Vishny (1993) show that value-investing strategies can generate higher returns, implying that the market is not perfectly efficient, i.e., there is a predictability that can be exploited [5].

To better profit in the stock market, primitive empirical analysis methods, traditional statistical methods, and artificial intelligence techniques have been used to analyze stocks. Empirical analysis methods, such as fundamental analysis, rely on analyzing the full spectrum of a stock over time. Traditional statistical methods such as linear regression and time series are difficult to adapt to complex stock analysis. For example, for the ARIMA model, Mehtabhorn Obthong et al. summarized its drawbacks, including unsuitability for nonlinear time series, and time-consuming nature when dealing with large-scale datasets [6]. Yang Li and Yi Pan argue that traditional methods are inadequate in dealing with complex stock market data, especially in capturing nonlinearities and dynamic changes [7]; Shizhan Liu et al. argue that traditional methods such as ARIMA and Prophet perform poorly in long-range prediction [8].

Artificial intelligence techniques have gradually been widely used by scholars as an emerging technology. Early machine learning techniques, such as decision trees, support vector machines, and clustering still have a number of drawbacks; for example, Firuz Kamalov argues that decision trees tend to overfit the data [9]; Ethem Alpaydin points out that the support vector machine method is computationally complex and difficult to deal with large-scale datasets, and that the final results of clustering algorithms are highly dependent on the selection of the initial clustering centers and are sensitive to noise and outliers [10]. Deep learning models show better performance than machine learning models, such as LSTM; RNN can handle complex nonlinear relationships and capture long-term dependencies in time series data, which is difficult for early machine learning methods [11]. Therefore, deep learning models, especially CNN and LSTM, are gradually being widely used for prediction in finance. Adil Moghara and Mhamed Hamiche used the LSTM model for stock market prediction and achieved good results, which verified the effectiveness of the LSTM model in stock market prediction [12]. Wen Long et al. proposed a multi-filter neural network (MFNN) model for stock prediction using six metrics, which demonstrated the advantages of deep learning methods over traditional methods [13]. However, there are still points to be improved in these methods, such as LSTM outperforming RNN in terms of performance but having a long running time [14]; Hadi Rezaei et al. point out that although models such as LSTM and CNN have yielded good results, the high volatility and stochastic nature of some time series make it still challenging to use these models alone [15]. Therefore, the attention on hybrid models is gradually increasing. Nan Jing et al. proposed to combine CNN and LSTM models with investor sentiment analysis, and the results showed the effectiveness of text data in stock prediction [16]; Xiaoqiao Huang et al. combined wavelet packet decomposition (WPD), convolutional neural network (CNN), long-short-term memory network (LSTM), and multilayer perceptron network (MLP) and verified their effectiveness on three datasets [17]. The findings of these studies suggest that the integration of deep learning models can significantly mitigate the limitations of individual models and enhance the precision of predictions. On the other hand, graph neural networks (GNNs) exhibit a notable capacity for relational dependency analysis. However, their reliance on well-defined graph structures imposes constraints that hinder their direct applicability to multivariate time series data. This hindrance contributes to the limited utilization of GNNs in related domains. For example, Karthigeyan Kuppan et al. combined the outputs of LSTM and GNN to obtain high $R^{2}$ scores and the lowest prediction errors [18]. In summary, the application of artificial intelligence to the domain of the stock market has emerged as a prevailing research trajectory within the academic community.

However, there are many imbalances in the relevant research. Firstly, most of the existing studies ignore the complex underlying relationships between stock data, i.e., the existence of mutual influence between companies; secondly, most studies use regression methods to predict the prices of individual stocks, and relatively few studies use categorization to predict the upward and downward trends of stocks. Third, most studies use individual types of characteristics in stock data as model inputs, and the analysis is relatively one-sided [19]. Fourthly, most of the existing studies are oriented to incremental markets such as the U.S. stock market, but there are few studies on stock markets such as A-shares, which are developing countries, and the volume–price relationships and model parameters applicable to incremental markets are not suitable for A-shares. In addition, among the few studies on A-shares, most of them focus on a few stocks, which is too limited in scope.

To address the above issues, this paper proposes a three-branch stock return prediction model for the A-share market, based on CNN, LSTM, and GNN. The primary contributions of this paper are as follows:

- A “Pearson and IG weighted selection” method is proposed to select features. This paper extracts the most suitable 5 features from 15 stock metrics that had been used in the study, i.e., daily return, turnover, RSI, volume, and qfq. After comparing the results of the actual model, the reasonableness of the features is verified. In addition, all the data used in this paper are the latest, real daily frequency trading data from the Cedar Capital and Tushare platform.
- A hybrid deep learning model based on CNN, LSTM, and GNN is proposed, analyzing the local features with CNN, the overall trend with LSTM, and the potential relationship between the data with GNN, which greatly improves the cumulative return after investing in the assets while reducing the confusion of the original model in selecting the highest return category. Subsequently, the proposed model is ablated with CNN, LSTM, CNN+LSTM model, single GNN model, CNN+GNN model, and LSTM+GNN model for the purpose of an ablation study. The model is then compared with a variety of other mainstream models to validate the effectiveness of the hybrid model.
- The stock pool is more selective and practical. This paper constructs portfolios in the entire A-share market of nearly 5000 stocks, with much greater selectivity than existing research. Moreover, the model proposed in this paper is an end-to-end model, which directly outputs the final recommended stock code and the return of the stock selection strategy.

The second chapter of this paper introduces the construction of the model, and the third chapter describes the experimental results. The fourth chapter discusses the results and points out the possible future research directions.

## 2. Methods

In this chapter, the method and process of feature selection, the architecture of the model, and the model’s processing of inputs are presented.

### 2.1. Feature Selection

The data used in this paper come from Cedar Capital and Tushare platform. Cedar Capital, founded in 2013, is an asset management company focusing on private securities investment business. Tushare is a financial data interface platform that provides API interfaces for all kinds of financial data, such as stocks, futures, funds, etc. The stock pool in this article is all normal stocks in the CSI All Share Index (CSI), except for those with insufficient listing time and ST special treatment.

In stock analysis, a multitude of optional data are included, which can be broadly classified into three primary categories: basic characteristics, technical indicators, and fundamental indicators. With regard to the basic characteristics, it includes data that reflect the basic trading conditions of the stock. Technical indicators are calculated based on data such as historical prices and volumes. These indicators are utilized for the analysis of stock price movements. Fundamental indicators focus on the financial condition and operating results of a company. Some of the features of the three categories are illustrated in Table 1.

There is obviously a great deal of redundant information in so many different types of historical data, and it is evident that incorporating such superfluous information into a model constitutes a suboptimal approach. Therefore, when applying deep learning models, feature selection is required to extract key features from the noisy data, neither too much nor too little. This process reduces the number of variables, lowers the computational cost, reduces the occurrence of overfitting and improves the model performance [20]. Htet Htet Htun et al. summarized the feature selection methods including filter, wrapper, and embedded methods, in which the filter method has the advantages of fast computation and robustness, but ignores the dependency relationship between features; the wrapper method can capture the complex relationship between features, but the computational cost is high and it is easily affected by overfitting; The embedded method combines the filter and wrapper methods, which is more efficient and less affected by overfitting [19]. Singh J and Khushi M pointed out through their study that technical indicators are not sufficient for long-term stock forecasting and the combination of basic characteristics and technical indicator data can make stock forecasting more accurate [21]. Therefore, this paper is based on the 15 kinds of stock data summarized by Zexin Hu et al. [22] that had been proved to be relatively effective by scholars’ research under the basic features and technical indicators, and uses the Pearson correlation analysis to analyze the correlation between each feature and the daily return, as well as the interrelationships among the features, and applies the Information Gain method for comparative validation, so as to select the appropriate input features.

Data for the 15 stock metrics selected for analysis are shown in Table 2, and abbreviations may be used in subsequent use.

Subsequently, the extended Pearson correlation coefficient and the correlation between the factors were calculated. The Pearson correlation coefficient is a statistical measure of the linear relationship between two variables. It is calculated using the following formula:

(1) $$r=\frac{\sum_{i=1}^{n}\left(x_{i}-\bar{x}\right)\left(y_{i}-\bar{y}\right)}{\sqrt{\sum_{i=1}^{n}{\left(x_{i}-\bar{x}\right)}^{2}\sum_{i=1}^{n}{\left(y_{i}-\bar{y}\right)}^{2}}}$$

The market value weights are introduced to calculate the weighted correlation coefficient, where the weighted mean is expressed as follows:

(2) $${\bar{x}}_{w}=\frac{\sum_{i=1}^{n}w_{i}x_{i}}{\sum_{i=1}^{n}w_{i}}$$

The formula for weighted covariance is

(3) $${\mathrm{Cov}}_{w}\left(x,y\right)=\frac{\sum_{i=1}^{n}w_{i}\left(x_{i}-{\bar{x}}_{w}\right)\left(y_{i}-{\bar{y}}_{w}\right)}{\sum_{i=1}^{n}w_{i}}$$

The formula for weighted variance is

(4) $${\mathrm{Var}}_{w}\left(x\right)=\frac{\sum_{i=1}^{n}w_{i}{\left(x_{i}-{\bar{x}}_{w}\right)}^{2}}{\sum_{i=1}^{n}w_{i}}$$

The weighted correlation coefficient is expressed as follows:

(5) $$r_{w}=\frac{{\mathrm{Cov}}_{w}\left(x,y\right)}{\sqrt{{\mathrm{Var}}_{w}\left(x\right){\mathrm{Var}}_{w}\left(y\right)}}$$

where the symbols are shown in Table 3.

For each stock in the stock pool, the correlation coefficients between its daily return and each factor are calculated after removing the missing values. These coefficients are then summarized after assigning weights based on the market value of the stock to obtain the average weighted correlation coefficient. Pearson correlation analysis was then performed between the factors in turn to identify possible multicollinearity and simplify the model. After removing highly correlated features (greater than 80%) and features with a correlation to return of less than 10%, 8 features are selected: daily return, RSI, amt, vol, turnover, qfq, mkt, close.

Subsequently, Information Gain (IG) is used for comparative validation. IG is the change in class entropy from a previous state to a known state and can be used to compute the correlation of features [23]. It further selects from the features obtained in the first step. For a feature X, the entropy(H) is

(6) $$H\left(X\right)=-\sum_{i=1}^{n}p\left(x_{i}\right)\log p\left(x_{i}\right)$$

Among them, $p(x_{i})$ is the probability that *X* takes the value $x_{i}$. The higher the entropy, the higher the degree of disorder of the data; the lower the entropy, the more orderly the data. Subsequently, the conditional entropy, i.e., the entropy of the feature X when given the variable Y, is calculated with the following formula:

(7) $$H\left(X|Y\right)=\sum_{y\in Y}p\left(y\right)H\left(X|Y=y\right)$$

Finally, the Information Gain is obtained, which is used to measure the gain resulting from feature segmentation, with the following formula:

(8) IG(T,X)=H(T)−H(T|X)

Among them, T is the target variable. A larger Information Gain indicates that the use of this feature for segmentation can significantly reduce the uncertainty of the system. Then, a subset of features is determined under the guidance of a set threshold value t.

Based on the Pearson correlation results, an IG model is constructed to calculate the correlation of each feature, and the top three features are obtained as follows: RSI, qfq, vol.

Combining the Pearson and IG results, as shown in the following equation:

(9) $$F_{o}-F_{hc}=F_{s}$$

(10) $$\mathrm{Result}=\mathrm{sort}\left(F_{s},\frac{I(F_{s})}{n}+\frac{R(F_{s})}{m}\right)$$

**Figure 1:**
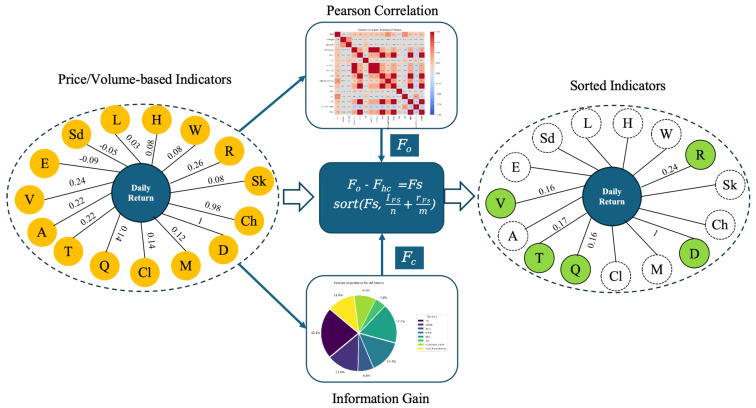
Feature selection process of “Pearson and IG weighted selection”.

$F_{o}$ is the original set of indicators, $F_{hc}$ is the highly correlated indicators, *n* and *m* are weights, and $I(F_{s})$ and $R(F_{s})$ are the Pearson correlation score and Information Gain score of features in the $F_{s}$ set, respectively. For all the indicators in the $F_{s}$ set, the correlations are calculated by equal weights, and sorted to obtain the final result. In summary, the feature selection method used in this paper is shown in Figure 1, and the final features obtained are daily return, turnover, RSI, vol, and qfq.

The relevant algorithm is shown in Algorithm 1:
**Algorithm 1** The Pearson and IG weighted selection pseudo-code.**Input**: $F_{o}$**Output**: Final features  1:df.fillna(value=pd.NA, inplace=True)  2:**for** each stock **do**  3:    $I\left(F_{s}\right)\leftarrow \mathrm{PearsonCorrelation}\left(F_{o},Dailyreturn\right)$  4:   $F_{hc}\leftarrow \mathrm{PearsonCorrelation}\left(F_{o},F_{o}\right)$  5:**end for**  6:$F_{s}=F_{o}-F_{hc}$  7:$R\left(F_{s}\right)\leftarrow \mathrm{IgImportance}\left(F_{s}\right)$  8:Final Features = np.sort(Fs + I(Fs) / n + R(Fs) / m)9:**return** Final Features
where qfq is similar to close, but qfq takes into account the decline in share price caused by factors such as corporate dividends on top of close. Most of the past research uses the original closing price as input consideration, but the compounded closing price has also been used by scholars in different scenarios and proved to be effective. For example, B. Xie et al. [24] use the adjusted closing price, which expands the application of natural language processing technology in financial market analysis by introducing a semantic framework and an innovative SemTree feature space. B. Voon Wan Niu et al. use daily adjusted closing price data over a period of about two decades to determine whether the Malaysian stock market exhibits chaotic characteristics [25]. Yumo Xu and Shay B. Cohen predicted stock moves by combining adjusted closing prices and text data from tweets [26]. This is not explored in depth in this paper and the case may be due to the existence of a delimitation gap in the original close, which may affect the training of the model, whereas the use of qfq may make the timing pattern captured by the LSTM consistent with the real investment returns.

### 2.2. Construction of Models

The model constructed in this paper is trained with data from the past 480 days and predicts the weekly returns for the following week using 60 days of data, and constructs a stock selection strategy by classifying stocks into five categories based on their returns and calculating the probability that each stock belongs to each category during a position change cycle (i.e., one week), thereby categorizing the stocks into the five categories of Surge, Rise, Flat, Fall, and Plummet. More accurately, the labels from 4 to 0 correspond to “Surge,” “Rise,” “Flat,” “Fall,” and “Plummet,” respectively, which indicate sharp increase, increase, nearly unchanged, decrease, and sharp decrease in stock trends. The model calculates the strategy returns of the selected stocks for each position change cycle and updates the stock picking strategy for each position change cycle by outputting the code of the selected stock.

**Figure 2:**
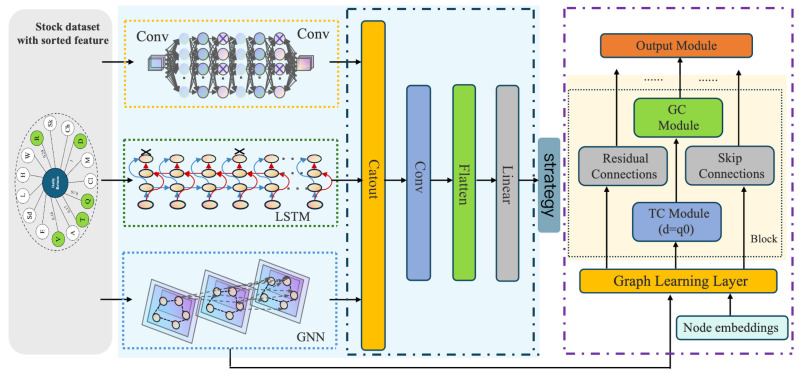
Framework of the CNN-LSTM-GNN model.

Firstly, the general framework of the CNN-LSTM-GNN neural network model proposed in this paper is explained. As demonstrated in Figure 2, the CLGNN model consists of a combination of CNN module, LSTM module, and GNN module.

Modern CNNs were laid down by Yann LeCun et al. and successfully used for handwritten digit recognition [27]. The convolutional algorithm that is activated by mish in this paper can be expressed as follows:

(11) y=mish(X×W+b)

where *X* is the input, *W* is the weight, *K* is the kernel size and *b* is the bias. Subsequently, Dropout is applied to improve the generalization of the model.

LSTM was proposed by Sepp Hochreiter and Jürgen Schmidhuber [28], and its algorithm can be expressed as follows.

(12) $$\begin{array}{cc} i_{t} & =\sigma (W_{ii}x_{t}+b_{ii}+W_{hi}h_{t-1}+b_{hi}) \end{array}$$

(13) $$\begin{array}{cc} f_{t} & =\sigma (W_{if}x_{t}+b_{if}+W_{hf}h_{t-1}+b_{hf}) \end{array}$$

(14) $$\begin{array}{cc} {\tilde{c}}_{t} & =\tanh(W_{ic}x_{t}+b_{ic}+W_{hc}h_{t-1}+b_{hc}) \end{array}$$

(15) $$\begin{array}{cc} c_{t} & =f_{t}⊙c_{t-1}+i_{t}⊙{\tilde{c}}_{t} \end{array}$$

(16) $$\begin{array}{cc} o_{t} & =\sigma (W_{io}x_{t}+b_{io}+W_{ho}h_{t-1}+b_{ho}) \end{array}$$

(17) $$\begin{array}{cc} h_{t} & =o_{t}⊙\tanh\left(c_{t}\right) \end{array}$$

where $i_{t}$ is the input to the input gate, $x_{t}$ is the input at time step *t*, $h_{t}$ is the hidden state at time step *t*, $c_{t}$ is the cell state at time step *t*, $f_{t}$ is the output of the forget gate, $o_{t}$ is the output of the output gate, ${\tilde{c}}_{t}$ is the candidate cell state, σ and tanh are the activation functions, *W* is the weight matrix, and *b* is the bias.

**Figure 3:**
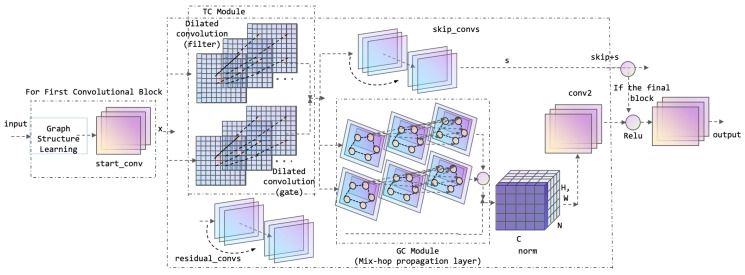
GNN module architecture.

Inspired by multivariate time series graph neural network [29], in this paper, MTGNN is modified as the basis of the GNN module as a module to analyze the potential connections between the data. The structure of the GNN module is illustrated in Figure 3:

As illustrated in Figure 3, the GNN module comprises a graph learning layer, n graph convolution blocks, n temporal convolution modules, and an output module. The graph learning layer is capable of computing and adapting the graph adjacency matrix in order to capture hidden relationships between time series data. In this paper, the assumption is made that the relationship between nodes is unidirectional, meaning that a change in one node affects some other node, rather than affecting each other in both directions. The nodes are embedded, and the adjacency matrix is ensured to be unidirectional by the following equation:

(18) $$M_{1}=\tanh\left(\alpha E_{1}\theta_{1}\right)$$

(19) $$M_{2}=\tanh\left(\alpha E_{2}\theta_{2}\right)$$

(20) $$A=\mathrm{Relu}(\tanh\left(\alpha \left(M_{1}M_{2}^{T}-M_{2}M_{1}^{T}\right)\right))$$

where $E_{1}$, $E_{2}$ are the initial node embeddings, $\theta_{1}$, $\theta_{2}$ are the model parameters, and α is the saturation rate of the activation function. When the value of $M_{1}M_{2}^{T}-M_{2}M_{1}^{T}$ is positive, it indicates that the influence of the previous node on the subsequent node is stronger than that of the subsequent node on the previous node; when the value is negative, the ReLU will truncate it to 0.

The time and graph convolution modules are interleaved so as to capture both temporal and spatial dependencies. In the time convolution module (TC), residual connections and jump connections are used to avoid gradient vanishing, which consists of a null convolution and inception layer, and the convolution kernel is chosen as 1×2, 1×3, 1×6, 1×7 to account for the natural temporal cycle.

In the graph convolution module, the information of nodes and neighbors is integrated through a process known as “mix-hop propagation,” which is comprised of two constituent components: information propagation and information selection. The specific formulation for this process is as follows:

(21) $$\begin{array}{cc} H^{\left(k\right)} & =\beta H_{\mathrm{in}}+\left(1-\beta \right)\tilde{A}H^{\left(k-1\right)} \end{array}$$

(22) $$\begin{array}{cc} H_{\mathrm{out}} & =\sum_{i=0}^{K}H^{\left(k\right)}W^{\left(k\right)} \end{array}$$

*K* is the propagation depth, $H_{\mathrm{in}}$ is the input hidden states outputted by the previous layer, $H_{\mathrm{out}}$ is the hidden output states of the current layer. The proportion of the node’s own state retention is regulated by β, and the neighbor information is propagated along the graph structure in a recursive manner; the important node features are filtered by the formula $H_{\mathrm{out}}=\sum_{i=0}^{K}H^{\left(k\right)}W^{\left(k\right)}$.

For the stock selection strategy optimization task, the model receives as input a three-dimensional tensor with dimensions expressed as (batch size, time step, feature dimension), i.e., each batch contains a batch size of stocks, each stock has time step days of data, and each day’s data have feature dimension individual features for each day. This input is processed through three distinct modules. The first module is the CNN module, which is composed of two convolutional blocks. The input passes through a 1D convolutional layer, a mish activation function layer, and a Dropout layer in sequence to generate the output of the CNN module. For the LSTM module, which contains a 2-layer LSTM network, a Dropout layer is applied between these two layers, and each LSTM unit processes the input data and transmits the hidden state to the subsequent layer, thereby contributing to the LSTM module output.

For the GNN module, the input first passes through the graph learning layer, and the sparse graph adjacency matrix is obtained based on the data computation, which is used as the input for the subsequent parts. The matrix first enters a starting convolutional layer and subsequently passes through three stacked convolutional blocks. The first convolutional block contains a TC module, a GC module, a residual convolutional layer, and a skip-joining convolutional layer. The inputs are first passed into the TC module and the copy of the input as residual. In the TC module, the input is passed into a filter dilation convolutional layer and gate dilation convolutional layer, respectively, using tanh activation function for filter and sigmoid activation function for gate to extract the feature information in the input data. Multiply the outputs of filter and gate and Dropout, and then pass into the skip-connected convolutional layer; in each convolutional block, the output of the skip-connected convolutional layer will be added with the previous skip-connected features, so that the features of different levels will be fused to avoid the loss of information in the process of passing. If the GC module has been enabled, the Skip is passed to the GC module concurrently, and the graph structure information is processed through the two mixprop graph convolution layers. Conversely, if the GC module has not been enabled, the ordinary residual convolution is performed on the output of the TC module. The output of the graph convolution or the residual convolution is summed up with the last dimension of the residual, thereby realizing the residual connection. Finally, it passes through the subsumption layer and is normalized. The output of the first block is passed through the same second block, and the resulting output is fed into the final convolution block, where all the skip-connect features are fused with the normalized output of the block. The fused features are then passed through the two ending convolution layers to obtain the output of the GNN.

The outputs of the three modules are spliced and subsequently passed through a 1D convolutional layer and mish activation function. This is then spread and passed through a linear layer to yield the final output.

## 3. Experiments

The models are tested empirically over the time horizon of 1 January 2024 to 1 April 2024, and shorting is not considered in all experiments. The cumulative returns in the experiments are the weekly returns of the selected stocks minus the relative cumulative returns of the combined returns of all stocks.

### 3.1. CLGNN

**Figure 4:**
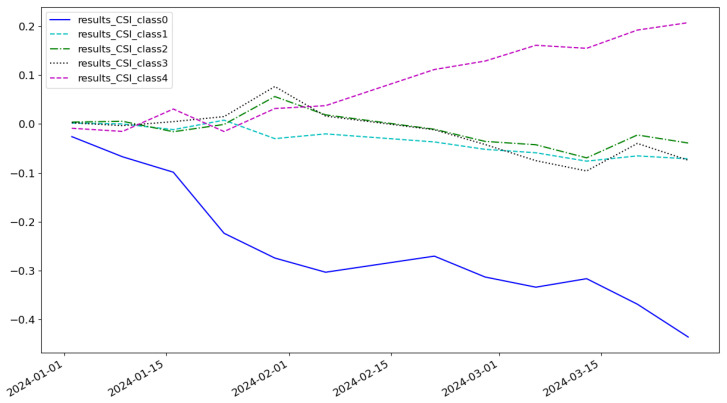
Cumulative return chart for CLGNN from 1 January 2024 to 31 March 2024.

Experimenting with the CLGNN model, as illustrated in Figure 4, the 3-month cumulative return can be as high as 20.7%, i.e., an annualized return of about 110%.

### 3.2. Ablation Study

A variety of features are utilized in distinct combinations as inputs, and the efficacy of these combinations is evaluated within a model employing identical hyperparameters. This process enables the validation of the selected parameters’ rationality. As a result, Table 4 is obtained. It is evident that distinct models possess unique parameters that are conducive to their particular applications. For instance, the Sd feature, which performs well in the original LSTM+CNN model, does not perform well in the CLGNN model. Moreover, the incorporation of the qfq feature results in a substantial enhancement of the yield in both models.

In order to show the superiority of the CLGNN model in A-share market prediction, LSTM, CNN, GNN model, dual-branching model combining CNN and GNN, and dual-branching model combining LSTM and GNN are introduced for an ablation study.

**Figure 5:**
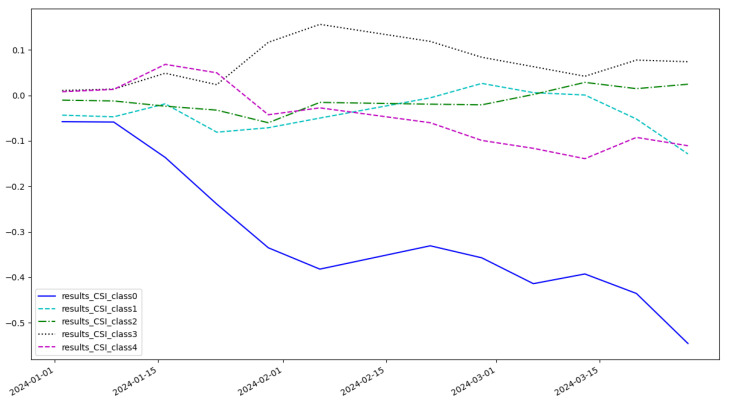
Cumulative return of LSTM model.

**Figure 6:**
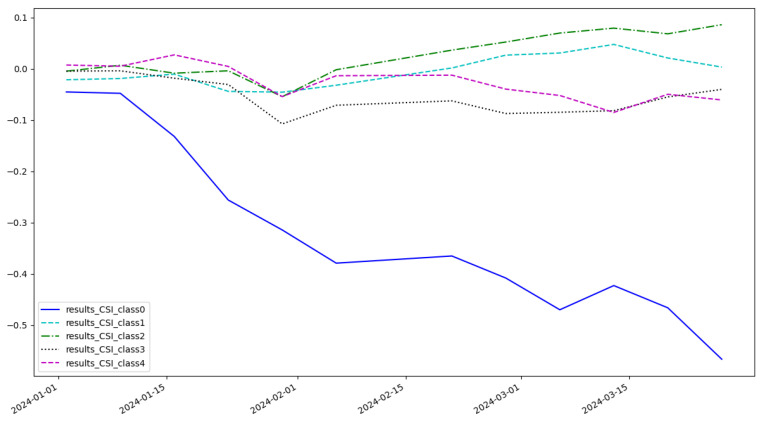
Cumulative return of CNN model.

**Figure 7:**
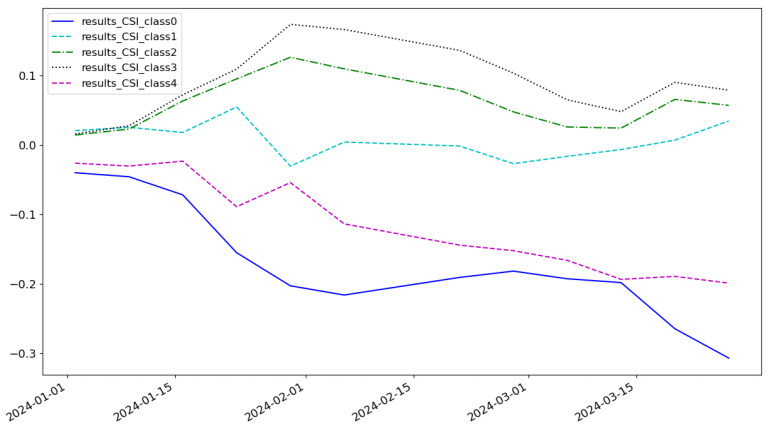
Cumulative return of GNN model.

**Figure 8:**
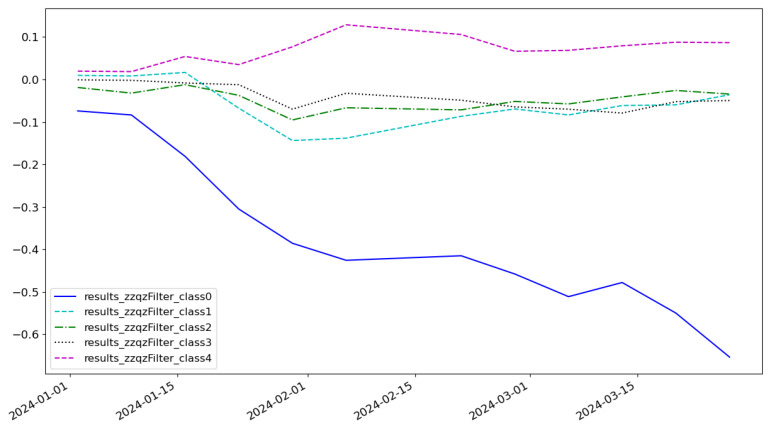
Cumulative return of CNN+LSTM model.

**Figure 9:**
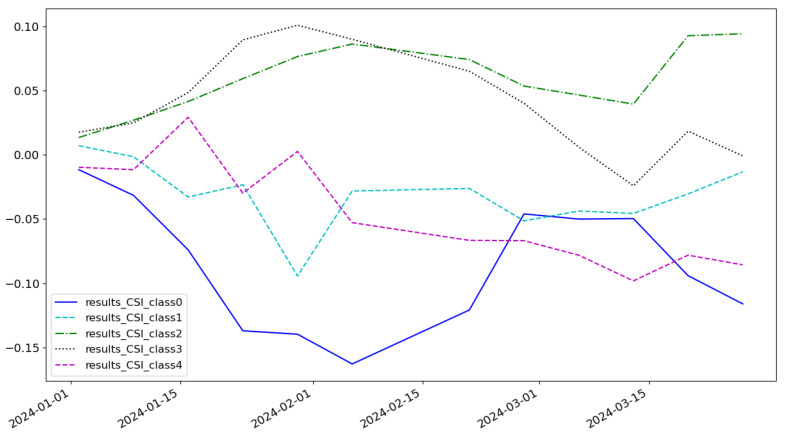
Cumulative return of CNN+GNN model.

**Figure 10:**
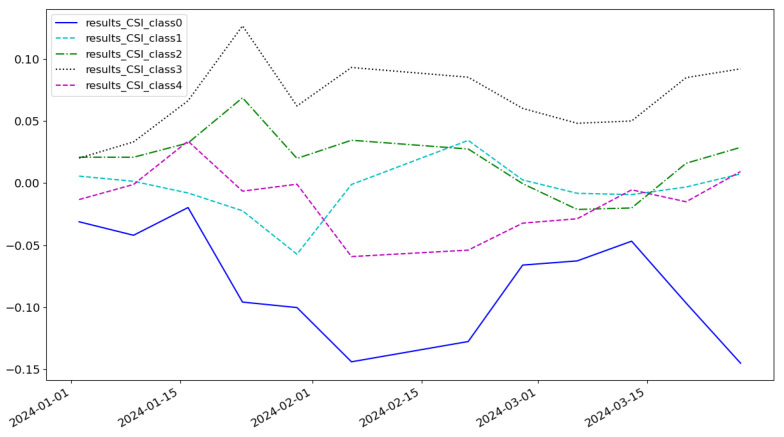
Cumulative return of LSTM+GNN model.

As shown in Figure 5, the cumulative return of the LSTM model is 0.074 and the highest category of return is class 3. As shown in Figure 6, the cumulative return of the CNN model is 0.086 and the highest category of return is class 2. As shown in Figure 7, the cumulative return of the GNN model is 0.079 and the highest category of return is class 4. As shown in Figure 8, the cumulative return of the CNN+LSTM model is 0.087 and the highest category of return is class 3. As shown in Figure 9, the cumulative return of the CNN+GNN model is 0.094 and the highest category of return is class 2. As shown in Figure 10, the LSTM+GNN model cumulative return is 0.092 and the highest category of return is class 3. It can be seen that the hybrid model outperforms the single model and the CLGNN model outperforms these models by 11.3% compared to the best of the remaining models, which is 0.094.

## 4. Results and Discussion

**Figure 11:**
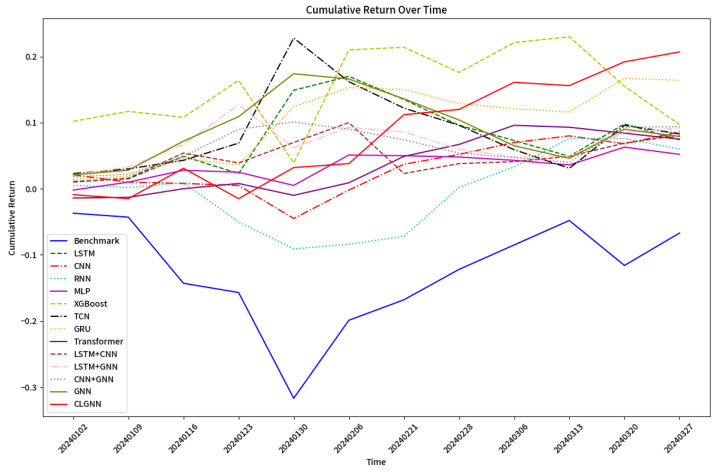
Summary charts of the returns of each model.

The highest return categories in the experimental results of each model are summarized in Figure 11 and Table 5. In addition, some statistical data for each model are summarized in Table 6.

**Figure 12:**
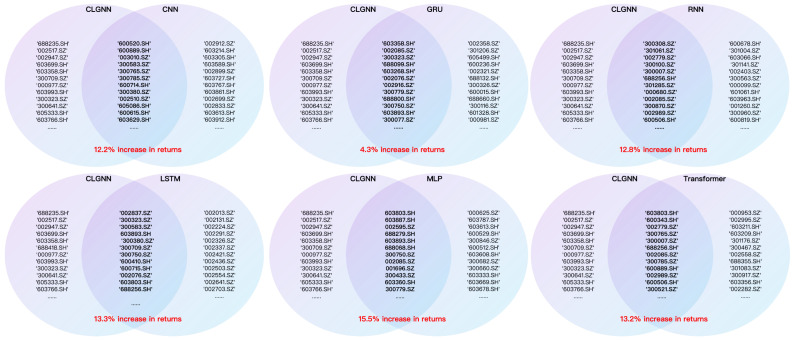
Stock code comparison chart (CLGNN model vs. other models).

The stock selection strategies derived from the CLGNN model are compared with other models, as shown in Figure 12. The final results are as follows:

- CLGNN has higher returns. The stock picking strategy suggested by CLGNN can obtain higher returns than the strategies proposed by other algorithms, and the cumulative returns remain up over time. Stock pools are more selective and practical.
- The stock selection strategy is different. In addition to the common recommended stocks, in the strategy proposed by CLGNN, the stocks belong more to industries in the growth period and policy support, such as current artificial intelligence, new energy, and so on. Other models, such as RNN, may favor traditional industries.
- qfq has excellent effects. In tests with different features for the same model, qfq as an input feature shows a significant increase in return.

## Figures and Tables

**Table 1:** Examples of some of the features of the three categories.

Feature Name	Categories	Feature Meaning
Market price	Basic characteristics	The first transaction price
Trading volume	Basic characteristics	Total number of shares traded
Market value	Basic characteristics	Fair market price
Moving average	Technical indicators	Weighted avg. for price trend
Relative strength index	Technical indicators	Measure buy/sell strength
Bollinger Bands	Technical indicators	Measure volatility and relative price
Debt-to-Asset ratio	Fundamental indicators	Debt burden relative to assets
Earnings per share	Fundamental indicators	Net profit divided by share capital
Price–earnings ratio	Fundamental indicators	Price per unit of earnings

**Table 2:** Symbol meaning table.

Data Name (Abbreviation)	Data Meaning
Open (O)	The first transaction price
Close (Cl)	The last transaction price
High (H)	The highest transaction price
Low (L)	The lowest transaction price
Amount (A)	The total value of stock transactions
Volume (V)	Number of transactions
turnover rate (T)	Share trading frequency
DailyRtns (D)	Return rate vs. the previous close
qfq (Q)	Adjusted closing price
mkt_freshares (M)	The total value of tradable shares
Change (Ch)	Diff. between current and previous close
Ema (E)	Weighted avg. for price trend
RSI (R)	Measures buy/sell strength
Stoch_k (Sk)	Checks overbought/oversold, % K
Stoch_d (Sd)	Checks overbought/oversold, % D
Williams (W)	Analyses overbought/oversold

**Table 3:** Table of meanings of symbols used in correlation calculations.

Data Name	Data Meaning
$x_{i}$	The *i*–th value of *x*
$y_{i}$	The *i*–th value of *y*
$w_{i}$	Weight of the *i*–th value
$\bar{x}$	Mean value of *x*
$\bar{y}$	Mean value of *y*
*n*	Number of values

**Table 4:** Feature combination effectiveness test.

Feature Combinations	Model	Cumulative Returns
D + T	LSTM + CNN	0.077
D + T + R	LSTM + CNN	0.090
D + T + V	LSTM + CNN	0.097
D + T + R + Sk	LSTM + CNN	0.062
D + T + R + V + Sk	LSTM + CNN	0.088
D + T + R + V + Sd	LSTM + CNN	0.138
D + T + R + V + Cl	LSTM + CNN	0.061
D + T + R + V + Q	LSTM + CNN	0.160
D + T + R + V + Sk + Sd	LSTM + CNN	0.083
D + T	CLGNN	0.108
D + T + R	CLGNN	0.168
D + T + V	CLGNN	0.172
D + T + R + Sk	CLGNN	0.140
D + T + R + V + Sk + Sd	CLGNN	0.096
D + T + R + V + Cl	CLGNN	0.173
D + T + R + V + Sd	CLGNN	0.090
D + T + R + V + Sk	CLGNN	0.158
**D + T + R + V + Q**	**CLGNN**	**0.207**

**Table 5:** Comparative table of model returns.

Models	Benchmark Return	Relative Return	Absolute Return
LSTM	−0.067	0.074	0.007
CNN	−0.067	0.085	0.018
RNN	−0.067	0.060	−0.007
MLP	−0.067	0.052	−0.015
XGBoost	−0.067	0.097	0.030
TCN	−0.067	0.083	0.016
GRU	−0.067	0.164	0.097
Transformer	−0.067	0.075	0.008
CNN+LSTM	−0.067	0.087	0.020
GNN	−0.067	0.079	0.012
CNN+GNN	−0.067	0.094	0.027
LSTM+GNN	−0.067	0.092	0.025
**CLGNN**	**−0.067**	**0.207**	**0.140**

**Table 6:** Model performance comparison.

Models	$F_{1}$	Loss	Return Rate
LSTM	0.282608	1.276278	0.074
CNN	0.301835	1.250813	0.085
RNN	0.259012	1.460925	0.060
MLP	0.231230	1.529214	0.052
XGBoost	0.328129	1.105234	0.097
TCN	0.294018	1.258410	0.083
GRU	0.502147	0.732809	0.164
Transformer	0.284788	1.272531	0.075
CNN+LSTM	0.309427	1.247536	0.087
GNN	0.288923	1.266902	0.079
CNN+GNN	0.314517	1.142936	0.094
LSTM+GNN	0.308695	1.170352	0.092
**CLGNN**	**0.647120**	**0.577703**	**0.207**

## Data Availability

The data presented in this study are available on request from the corresponding author.
